# Compositional associations of 24-h physical activities, sedentary time and sleep with depressive symptoms in urban and rural residents: a cross-sectional study

**DOI:** 10.1186/s12916-025-04051-9

**Published:** 2025-04-14

**Authors:** Marjo Seppänen, Tiina Lankila, Maisa Niemelä, Nina Rautio, Maija Korpisaari, Markku Timonen, Raija Korpelainen, Vahid Farrahi

**Affiliations:** 1 Geography Research Unit, University of Oulu, Oulu, Finland; 2 Research Unit of Population Health, University of Oulu, Oulu, Finland; 3 Department of Sports and Exercise Medicine, Oulu Deaconess Institute Foundation sr., Oulu, Finland; 4 Research Unit of Health Sciences and Technology, University of Oulu, Oulu, Finland; 5 Centre for Wireless Communications, University of Oulu, Oulu, Finland; 6 Unit of General Practice, Oulu University Hospital, Oulu, Finland; 7 Medical Research Center Oulu, Oulu University Hospital and University of Oulu, Oulu, Finland; 8 Institute for Sports and Sport Science, TU Dortmund University, Dortmund, Germany

**Keywords:** Compositional data analysis, Depressive symptoms, Urban, Rural, Physical activity, Sedentary time, Sleep

## Abstract

**Background:**

Studies investigating the associations of 24-h movement behaviours (including moderate-to-vigorous-intensity physical activity (MVPA), light-intensity PA (LPA), sedentary time (ST) and sleep) with depressive symptoms are scarce. It is also unclear whether possible associations differ between urban and rural residents. Hence, we aimed to investigate these associations in a population-based sample of middle-aged Finnish adults.

**Methods:**

The study population consisted of 4295 adults, aged 46 years, from the Northern Finland Birth Cohort 1966. The participants wore a hip-worn accelerometer for 14 days. Time spent in sedentary, LPA and MVPA was obtained from accelerometer data and then combined with self-reported sleep duration to obtain the 24-h composition. The residential environment was classified as urban or rural based on the participants’ home addresses. Depressive symptoms were assessed using the Beck Depression Inventory-II (BDI-II). Multivariable adjusted regression analysis using a compositional data analysis approach based on isometric log-ratio transformation was used to determine the associations between movement behaviours and depressive symptoms in urban and rural residential environments.

**Results:**

The 24-h movement behaviour composition was significantly associated with the BDI-II score both in urban and rural residential environment. More time spent in sleep relative to other behaviours was associated with lower BDI-II score in rural residential environments. More time spent in ST among urban residents and in LPA among rural residents was associated with higher BDI-II scores. When modelling pairwise reallocations of time, more MVPA or more sleep at the expense of LPA or ST was associated with lower BDI-II score among rural residents. For urban residents, reallocating time from ST to any other behaviour was associated with lower BDI-II score.

**Conclusions:**

Our findings showed that more relative time spent in MVPA and sleep was associated with lower levels of depressive symptoms among rural residents, and more relative time spent in any other behaviour at the expense of ST was associated with lower levels of depressive symptoms among urban residents. These differences should be considered in the prevention and treatment of depressive symptoms. Due to the cross-sectional design of this study, causality cannot be inferred, and further research exploring the mechanisms underlying these associations in diverse populations and longitudinal study settings are needed.

**Supplementary Information:**

The online version contains supplementary material available at 10.1186/s12916-025-04051-9.

## Background

Depression is the leading cause of disability worldwide, affecting over 4.4% of the global population [1]. Its prevalence along with the associated costs is increasing [2], and the recent COVID- 19 pandemic has further exacerbated psychological distress [3]. Therefore, understanding the behavioural and environmental factors associated with depression and depressive symptoms is crucial.

One significant behavioural factor associated with depression is physical activity (PA). A systematic review suggested that PA can prevent future depression [4]. A more recent systematic review and network meta-analysis [5] found exercise to be an effective treatment for depression. Similar findings were reported in an umbrella review [6], which provided compelling evidence of an association between PA and lower levels of depression and highlighted the importance of engaging in multimodal, moderate-to-vigorous-intensity PA (MVPA). Even in individuals without clinical depression, exercise-based interventions have been shown to reduce depressive symptoms [7].

A full 24-h day includes time spent in MVPA, light-intensity PA (LPA), sedentary and sleep, collectively referred to as 24-h movement behaviours. Evidence suggests that both insufficient and excessive sleep [8] as well as higher amounts of mentally passive sedentary time (ST) [9] may increase the risk of depression. However, studying movement behaviours in isolation does not take into account the finite nature of the 24-h day, or that higher time spent on one behaviour inevitably means less time spent on, at least, one other behaviour [10]. To better understand the role of different movement behaviours on health, compositional data analysis has emerged as a novel technique in PA research, allowing for the consideration of all movement behaviours in relation to one another [10].

Few studies have used compositional analysis to determine the associations between movement behaviours and depressive symptoms. Blodgett et al. [11] utilised compositional data analysis to investigate the relationship between PA, sedentary behaviour, sleep and depression risk in the 1970 British Cohort study. Their findings demonstrated that reallocating time from any other behaviour to MVPA was associated with a lower risk of depression, whereas reallocating time between other behaviours had minimal or no effect. Similar studies found that reallocating time from ST to sleep [12, 13] and to LPA [13], in addition to increased MVPA, is associated with decreased depressive symptoms.

With an increasing number of people living in urban areas [14], it is important to consider the role of urbanicity (or ‘the impact of living in urban areas’ [15]) of the residential environment on PA and depressive symptoms. Although urbanicity has been associated with a higher risk of depressive symptoms in Finland [16], this association appears to be more complex globally [17]. To date, findings on the relationship between urbanisation and the incidence of depression remain inconsistent. Studies conducted in various countries reported most often either an association between higher urbanisation and depressive mood or no association at all [18]. In addition, it was suggested that individuals living in rural environments engage in higher levels of total PA, probably due to household activities, and urban residents (especially in high-income countries) participate more in high-intensity activity [19] and recreational PA [20]. Evidence further suggests that changes in the built environment, such as improved accessibility and new infrastructure for walking, cycling and public transport, may be associated with higher PA levels [21].

Given the lack of previous studies considering both 24-h movement behaviour time use and the urbanicity of the living environment in relation to depressive symptoms, our study aimed to address this gap. We investigated whether the 24-h movement behaviour composition is associated with the BDI-II score among urban and rural residents using a compositional data analysis approach and examined how the reallocation of time among 24-h movement behaviours in those living in urban and rural areas is associated with the BDI-II score.

## Methods

### Study population

This cross-sectional study used data from the Northern Finland Birth Cohort 1966 (NFBC1966) study, a longitudinal research programme [22] that originally included all individuals whose expected birth was in 1966 in Northern Finland (*n* = 12,058 live-born individuals) [23]. Cohort members were followed from the prenatal period onwards by interviews, postal questionnaires and clinical measurements. This study analysed the data from the most recent time point when the individuals were 46 years old, with a target population of 10,321. Of the total target population, 5832 cohort members participated in the clinical examination. Eligible participants for this study were those cohort members who completed the Beck Depression Inventory-II (BDI-II) questionnaire, provided valid accelerometer data, and had valid address information to define urban/rural residency, resulting in 4295 eligible participants for the present study. Details on the cohort profile, including the loss of follow-up and missingness, have been previously reported elsewhere [24]. NFBC1966 was approved by the Ethical Committee of the Northern Ostrobothnia Hospital District (94/2011), and written informed consent was obtained from all participants.

### Measurements

#### Twenty-four hour movement behaviours

Participants were asked to wear a hip-worn accelerometer (Hookie AM20; Traxmeet Ltd., Espoo, Finland) for 14 consecutive days during all waking hours, except for water-based activities. Raw acceleration signals were collected and stored at 100 Hz. The accelerometer data were first segmented into 6-s epochs, and mean amplitude deviation (MAD) values were computed for each 6-s epoch [25]. The MAD values of Hookie demonstrate excellent agreement with the ActiGraph GTX3, which is one of the most widely used accelerometers in PA research [26]. Previous studies have generally used 5-s epochs to estimate ST, LPA and MVPA, typically without consideration of anatomical posture and only on the basis of energy expenditure. In our approach, we used 6-s epochs to allow further differentiation between standing still and sitting or lying using a previously validated method [27]. This method was developed and validated using 6-s epochs to achieve finer granularity for further differentiation between standing still and sitting or lying. From the 6-s epochs, accelerometer non-wear intervals were identified and removed using a widely adopted method for count-based data, with a 30-s threshold to manage artifactual acceleration [28].

The detected wear-time intervals were then cross-referenced with self-reported sleep times (captured with two questions: ‘At what time do you normally go to bed?’ and ‘At what time do you normally get out of bed?’). All accelerometer data that overlapped with a sleep interval were discarded. The remaining 6-s epochs were classified as sedentary (sitting or lying), standing still, LPA, or MVPA based on MAD values [27, 29], and minutes per day in each activity was obtained by dividing time spent in each activity by the number of valid days. Further differentiation between standing still and sitting or lying was performed using a previously validated method [27]. This approach enables posture estimation from hip-based raw acceleration data based on constant Earth’s gravity vector and upright walking posture. This method has shown good to excellent accuracy when compared with thigh-worn posture classification as ground truth under free-living conditions [27]. For the purposes of this study, LPA constituted the sum of all minutes per day spent standing still and in LPA. [27, 29] To be included in this study, participants had to provide at least four valid days of accelerometry data, with each valid day defined as wearing the monitor for ≥ 10 h. Sleep duration was self-reported by answering the question, ‘How many hours do you sleep on average per day?’. Answers were then converted to minutes asleep per day.

#### Urban–rural classification

The Finnish Environment Institute’s urban–rural classification divides Finland into seven different categories: (1) inner urban areas, (2) outer urban areas, (3) peri-urban areas, (4) local centres in rural areas, (5) rural areas close to urban areas, (6) rural heartland areas and (7) sparsely populated rural areas [30, 31]. The urban–rural classification is based on geospatial methods and data, including information on population, workforce, commuting and buildings, as well as road network data and CORINE land use data. The data represents the year 2010. We linked the urban–rural classification data to the home coordinates of the cohort members. Of those with valid accelerometer data and completed BDI-II questionnaire, urban/rural residency could not be defined for 10 (0.2%) participants due to missing coordinates. For the analyses, categories 1–3 were combined and classified as urban areas and categories 4–7 as rural areas.

#### Depressive symptoms

The cohort participants completed the 21-item BDI-II questionnaire, indicative of depressive symptoms, on the day of their health examination. They responded to each item on a scale of 0–3, with higher cumulative scores indicating a greater severity of depressive symptoms. The total score of the BDI-II can be divided into the following categories: no depressive symptoms (scores 0–13), mild depressive symptoms (14–19), moderate depressive symptoms (20–28) and severe depressive symptoms (26–63) [32]. In our study population, the Cronbach’s alpha coefficient for the BDI-II items was found to be 0.91, indicating excellent internal consistency. Of those with valid accelerometer data, 4% (*n* = 184) did not complete the BDI-II questionnaire.

#### Covariates

Covariates were selected based on previous literature and preliminary analyses. Medical records were used to extract sex. The participants provided information on their education, employment, strenuousness of work (scores 0–9, based on 9 items described in detail previously by Punakallio et al. [33]) and marital status. Smoking status was classified as non-smoker, former smoker or current smoker. Alcohol consumption was based on questions about the frequency of use and typical quantity per occasion of (i) mild drinks, (ii) wines and (iii) spirits. For each drink, we multiplied the frequency by the amount to calculate the daily consumption of alcohol in grams, as described in detail previously by Vladimirov et al. [34]. Participants also answered the Cloninger’s Temperament and Character Inventory, from which information on the harm avoidance personality trait summary score was retrieved and included in the models, as it is associated with depression [35]. Waist circumference (cm) was measured during the clinical examination. As the time spent in different movement behaviours varies over the seasons [36], the season of the year (summer or winter) of the accelerometer data collection was also added as a covariate.

### Statistical analyses

Analyses were performed for participants with complete data with no missing values for covariates, exposures and outcome variable (*n* = 3498). Analyses were stratified based on residential environment into urban (*n* = 2364) and rural (*n* = 1134) areas. Standard descriptive statistics (counts, proportions, means and standard deviations [SDs]) were used to describe the participants’ characteristics. For the 24-h movement behaviours, compositional means—calculated by scaling the geometric mean of each behaviour to sum to 24 h—are presented, as they are more representative of compositional data. Standard statistical tests are not suitable for assessing group differences in 24-h time-use data [37]. Following previously published recommendations for examining group differences in such data [37], the differences in MVPA, LPA, ST and sleep between urban and rural residents were assessed by computing the log-ratio of the geometric mean values of these behaviours in urban and rural residents. We used bootstrapping with 1000 replicates and replacements to obtain a 95% confidence interval (CI).

Multivariable adjusted regression analysis, using a compositional data analysis approach based on isometric log-ratio transformation [38, 39], was used to examine the associations among 24-h movement behaviours with the BDI-II score among urban and rural residents. The association between sleep and depressive symptoms could possibly be U-shaped, with both short and long durations exhibiting adverse associations with depression symptoms [40]. We examined the possible U-shaped association between sleep and BDI-II score using a two-lines test [41]. The two-lines test has shown to be a robust test for assessing U-shapedness. We found no signs of a U-shaped relationship between sleep duration and BDI-II score (see the Additional File 1: Fig. S1). In addition, we estimated how the pairwise time reallocations from one movement behaviour to another were associated with the estimated change in the BDI-II score [39]. Pairwise time reallocations among movement behaviours ranged from 10–60 min, with a 10-min increment. Time reallocations to/from MVPA were constrained to 30-min to remain below the sample mean for MVPA. The results of the pairwise time reallocation analyses were plotted, displaying the percentage change in the mean value of the BDI-II, along with the corresponding 95% CI [39].

As a sensitivity analysis, and to confirm the robustness of our results, we repeated the analyses described above for participants who slept between 7 and 9 h per night, which is the recommended amount of sleep for adults [42]. For all analyses, statistical significance was set to 0.05. All statistical analyses were performed using R [43] and R Studio [44]. Packages robCompositions [45] and compositions [46] were used to perform compositional data analyses, packages tidyverse [47] and gridExtra [48] to create the plots and package Table 1 [49] to create the characteristics table.

**Table 1:** Characteristics of the whole sample and urban (*n* = 2868) and rural (*n* = 1427) residents by depressive symptoms

	All (*n* = 4295)	Urban residents (*n* = 2868)	Rural residents (*n* = 1427)
**Sex**			
Male	1833 (42.7%)	1204 (42.0%)	629 (44.1%)
Female	2462 (57.3%)	1664 (58.0%)	798 (55.9%)
Missing	0 (0%)	0 (0%)	0 (0%)
**Highest education**			
Comprehensive school	147 (3.4%)	71 (2.5%)	76 (5.3%)
Vocational- or college-level education	3121 (72.7%)	1966 (68.5%)	1155 (80.9%)
Polytechnic or university degree	1025 (23.9%)	830 (28.9%)	195 (13.7%)
Missing	2 (0%)	1 (0%)	1 (0.1%)
**Employment**			
Employed	3521 (82.0%)	2383 (83.1%)	1138 (79.7%)
Unemployed	187 (4.4%)	114 (4.0%)	73 (5.1%)
Other (e.g. student or homemaker)	514 (12.0%)	323 (11.3%)	191 (13.4%)
Missing	73 (1.7%)	48 (1.7%)	25 (1.8%)
**Strenuousness of work**	4.0 (3.0)	3.6 (2.9)	4.8 (2.9)
Missing	537 (12.5%)	329 (11.5%)	208 (14.6%)
**Marital status**			
Married or cohabiting	3403 (79.2%)	2207 (77.0%)	1196 (83.8%)
Divorced or widowed	426 (9.9%)	323 (11.3%)	103 (7.2%)
Unmarried	451 (10.5%)	332 (11.6%)	119 (8.3%)
Missing	15 (0.3%)	6 (0.2%)	9 (0.6%)
**Harm avoidance personality trait**	13.1 (6.3)	12.9 (6.3)	13.6 (6.3)
Missing	260 (6.1%)	170 (5.9%)	90 (6.3%)
**Alcohol consumption (g/day)**	10.5 (17.0)	11.3 (17.4)	8.87 (16.0)
Missing	2 (0%)	0 (0%)	2 (0.1%)
**Smoking status**			
Non-smoker	2317 (53.9%)	1535 (53.5%)	782 (54.8%)
Former smoker	1167 (27.2%)	791 (27.6%)	376 (26.3%)
Current smoker	772 (18.0%)	516 (18.0%)	256 (17.9%)
Missing	39 (0.9%)	26 (0.9%)	13 (0.9%)
**Waist circumference (cm)**	91.3 (13.5)	90.8 (13.2)	92.3 (14.0)
Missing	24 (0.6%)	18 (0.6%)	6 (0.4%)
**Season of the PA data collection**			
Summer	2324 (54.1%)	1462 (51.0%)	862 (60.4%)
Winter	1971 (45.9%)	1406 (49.0%)	565 (39.6%)
Missing	0 (0%)	0 (0%)	0 (0%)
**Mean accelerometry wear time (min/day)**	854 (59.0)	854 (58.6)	855 (59.8)
Missing	0 (0%)	0 (0%)	0 (0%)
**BDI-II score**	5.3 (6.1)	5.3 (6.2)	5.3 (5.8)
Missing	0 (0%)	0 (0%)	0 (0%)

Continuous variables are presented as means (SD) and categorical variables as counts (%)

*BDI-II *Beck Depression Inventory-II,* SD *standard deviation

## Results

Detailed descriptive statistics of the participants included in the analyses are presented in Table 1. Of the total 4295 participants, 57.3% were females. The mean BDI-II score for the total population was 5.3 (SD = 6.1), and it was the same among urban (SD = 6.2) and rural (SD = 5.8) residents.

Participants living in urban residential environments were more highly educated, were more likely to be employed and had a smaller waist circumference than those living in rural residential environments. Rural residents were more likely to be married, consume less alcohol and be non-smokers than urban residents. PA data from rural residents were more often collected during the summer.

### Main analysis

**Fig. 1:**
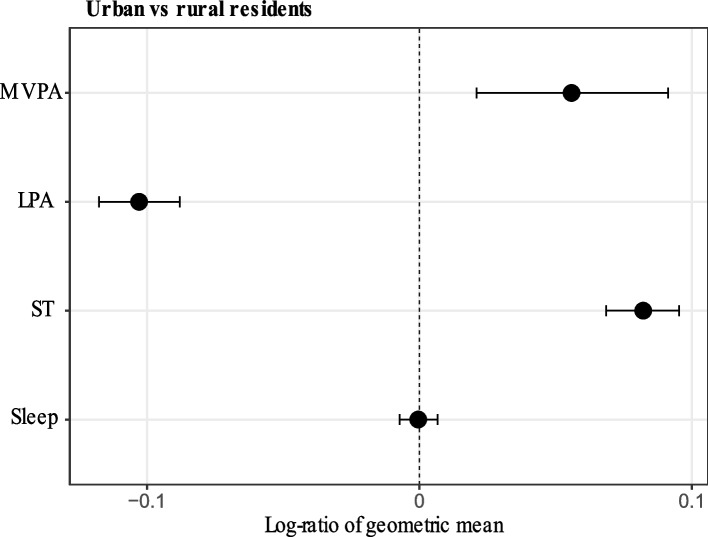
Differences with 95% bootstrap percentile CIs between urban and rural residents in the log-ratio of geometric mean values for MVPA, LPA, ST and sleep in all eligible participants (*n* = 4295). Notes: Urban was used as the numerator and rural as the denominator when calculating the log-ratios. A positive value of the log-ratio indicates that urban residents spent more time in that behaviour compared to rural residents, whereas a negative value indicates that urban residents spent less time in that behaviour compared to rural residents. A particular behaviour is considered significantly different between groups if its CI does not include zero. MVPA, moderate-to-vigorous-intensity physical activity; LPA, light-intensity physical activity; ST, sedentary time; CIs, confidence intervals

The compositional means of movement behaviours, for the whole sample and stratified by urban/rural residency, are shown in Additional File 1: Table S1. Figure 1 demonstrates the differences in each component of 24-h movement behaviours between those living in urban and rural areas. Participants living in urban residential environments had higher amount of MVPA (0.06, 95% CI: 0.02, 0.09) and ST (0.08, 95% CI: 0.07, 0.10), but lower amount of LPA (− 0.10, 95% CI: − 0.12, − 0.09) than those living in rural residential environments. No statistically significant differences in sleep were observed.

The results of compositional regression analysis are presented in Table 2. The composition of movement behaviours across the 24-h day was significantly associated with the BDI-II score regardless of the urban/rural residency (model *p*-value < 0.05 for all). For the whole sample, more daily time in ST in relation to other behaviours was associated with higher BDI-II score, whereas more sleep was associated with lower BDI-II score after accounting for the confounders. For urban residents, more ST in relation to other behaviours was associated with higher BDI-II score, and for rural residents, more time spent in LPA was associated with higher BDI-II score and sleep with lower BDI-II score.

**Table 2:** Compositional multiple linear regression estimates for BDI-II score by depressive symptom status and urban/rural residency

	**Model *R*2**	**Model *p***	**MVPA *β* (95% CI)**	***p***	**LPA *β* (95% CI)**	***p***	**ST *β* (95% CI)**	***p***	**Sleep *β* (95% CI)**	***p***
All (*n* = 3498)	0.23	** < 0.001**	− 0.20 (− 0.56–0.16)	0.284	*0.68 (*− *0.09–1.46)*	*0.083*	**1.29 (0.46–2.11)**	**0.002**	** − 1.77 (− 2.98 to − 0.56)**	**0.004**
Urban residents (*n* = 2364)	0.24	** < 0.001**	− 0.12 (− 0.57–0.33)	0.596	− 0.11 (− 1.07–0.85)	0.824	**1.29 (0.25–2.33)**	**0.015**	− 1.06 (− 2.57–0.46)	0.171
Rural residents (*n* = 1134)	0.23	** < 0.001**	− 0.47 (− 1.09–0.14)	0.131	**2.42 (1.11–3.72)**	** < 0.001**	*1.25 (*− *0.13–2.63)*	*0.076*	** − 3.19 (− 5.21 to − 1.17)**	**0.002**

Significant associations at level *p*<0.05 are shown in bold, and associations at level *p*<0.10 in italic. All the models have been adjusted for sex, education, employment status, strenuousness of work, marital status, harm avoidance personality trait score, alcohol consumption, smoking status, waist circumference and the season of the PA data collection

*MVPA *moderate-to-vigorous-intensity physical activity,* LPA *light-intensity physical activity,* ST *sedentary time

### Time reallocations

**Fig. 2:**
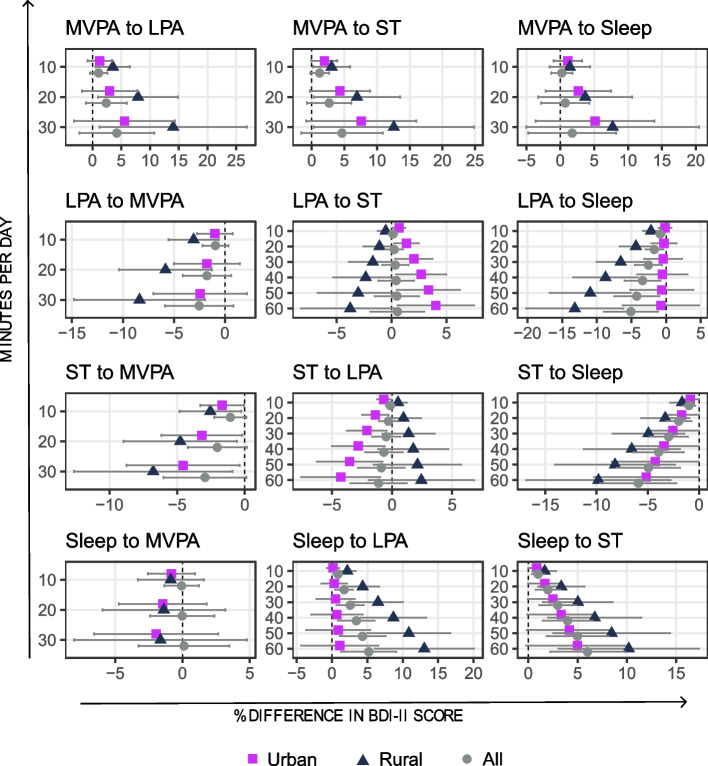
Estimated %differences (95% CI) in BDI-II score from pairwise time reallocations between movement behaviours among the urban (*n* = 2364), rural (*n* = 1134) and total population (*n* = 3498). Note that the scale of the *x*-axis varies. BDI-II, Beck Depression Inventory-II

Figure 2 shows the results of the pairwise time reallocations from all movement behaviours to others for urban and rural residents and for the whole population. Statistically significant associations were observed for urban and rural residents and the total population. Among urban residents, reallocating time from ST to any other behaviour was associated with lower BDI-II score. For example, reallocating 30 min from ST to MVPA or sleep was associated with a − 4.6% (95% CI: − 8.7, − 0.4) and a − 2.5% (95% CI: − 5.1, − 0.002) lower BDI-II score, respectively. In addition, reallocating time from LPA to ST was associated with a higher BDI-II score.

In rural residents, reallocating 30 min from LPA or ST to MVPA was associated with a − 8.4% (95% CI: − 14.8, − 2.0) and − 6.8% (95% CI: − 12.6, − 0.9) lower BDI-II score, respectively. Furthermore, reallocating 60 min from LPA or ST to sleep was associated with a − 13.2% (95% CI: − 20.2, − 6.2) and − 9.8% (95% CI: − 16.9, − 2.8) lower BDI-II score among rural residents, respectively. Conversely, reallocating time from MVPA to LPA or ST, or from sleep to LPA or ST, was associated with a higher BDI-II score.

In the total population, reallocating 60 min from LPA or ST to sleep was associated with a − 5.1% (95% CI: − 9.0, − 1.1) and − 5.9% (95% CI: − 9.6, − 2.2) lower BDI-II score, respectively. Conversely, reallocating 60 min from sleep to LPA or ST was associated with a 5.2% (95% CI: 1.3, 9.1) and a 6.0% (95% CI: 2.2, 9.8) higher BDI-II score.

### Results from the sensitivity analysis

When the analyses were repeated for participants with healthy sleep duration (7–9 h per night, *n* = 2874), similar association patterns to the main analyses were observed especially among rural residents. The main differences were that the time spent in sleep and in sedentary among total population and the time spent in sedentary among urban residents were not statistically significant. See Additional File 1: Table S2, for detailed results of the compositional regression analysis.

## Discussion

This cross-sectional study investigated the associations between the composition of 24-h movement behaviours and depressive symptoms in adults living in urban and rural residential environments. The main finding was that among urban residents, reallocating time from ST to any other behaviour was associated with lower BDI-II score, and among rural residents, more MVPA at the expense of LPA or ST or more sleep at the expense of LPA or ST was associated with lower BDI-II score.

This is the first study to use a compositional data analysis approach to model the association between movement behaviours and depressive symptoms while taking into account residential environment. In Finland, urbanicity is reportedly associated with an increased risk of depressive symptoms [16], although the association between the urban environment and depressive symptoms in general is controversial [18]. In this study, we found that more time spent in MVPA at the expense of ST was beneficial for urban residents, and more time spent in MVPA at the expense of LPA or ST was beneficial for rural residents. The mean amount of MVPA was significantly lower among rural residents, which might explain why the higher time spent in MVPA was even more beneficial among them. Several previous studies using the compositional data analysis approach have also found that higher time spent in MVPA at the expense of any other behaviour [11], especially at the expense of ST [12, 13], is the most beneficial time reallocation for improving depressive symptoms. Mechanisms behind the beneficial effects of PA on depressive symptoms include stimulation of neuroplastic processes involved in depression, reduction of inflammation and increased resilience to oxidative and physiological stress [50], and lower-intensity PA may be insufficient to activate these mechanisms [51].

Although there is increasing evidence that, in addition to MVPA, adults may also gain health benefits from LPA [52–54], our findings indicate that more time spent in LPA at the expense of MVPA or sleep was associated with higher BDI-II score among rural residents. However, among urban residents, more time spent in LPA at the expense of ST was associated with lower BDI-II score. Previous studies using a compositional data approach to investigate the association between LPA and depressive symptoms have reported marginal beneficial associations [11, 13] or no association at all [12]. In our study, the amount of LPA was significantly higher among rural residents, which might partly explain our results. Overall, similar urban–rural differences in PA have been previously reported, with rural residents reporting more total PA [19, 20] and urban residents more high-intensity PA [19]. As suggested in earlier studies, the main differences in PA levels are due to higher amounts of household and occupational PA in rural areas [19, 20] and higher amounts of recreational PA in urban areas, which is especially typical in high-income countries [20] such as Finland. The context of LPA may explain the results of our study. Previous research on context-specific PA has shown that work-related PA could unfavourably influence mental health, whereas household PA has no significant association with mental health [55]. Although we could not distinguish the domain of PA in our study, both the intensity and context of PA may have a role in reducing depressive symptoms. This is supported by psychosocial and behavioural mechanisms, such as improved self-esteem, self-efficacy, and increased social support, which are suggested to contribute to the beneficial effects of PA on depression [50].

Our results regarding ST were consistent with previous studies [9, 12, 13], which found that higher time spent in ST was associated with higher depressive symptoms. We found that lower BDI-II score was associated with higher relative time spent in any other behaviour at the expense of ST among urban residents. Reallocating time from ST to MVPA or sleep was also associated with lower BDI-II score among rural residents. The mean amount of ST was significantly higher among urban residents, contrary to the previous review reporting lower ST levels in urban areas [56]. This could be attributed to the different methodologies used in ST measurement, as most of the studies included in the review were based on self-reported ST.

Reallocating more daily time to sleep at the expense of ST was associated with lower BDI-II score in both urban and rural residents, and for rural residents, also reallocating more time to sleep at the expense of LPA was associated with lower BDI-II score. This is similar to the findings of Cruz et al. [12] and Kandola et al. [13], who found that more time spent in sleep at the expense of ST was associated with lower depressive symptoms. On the other hand, Blodgett et al. [11] found no association between sleep time and depressive symptoms, which might be due to the higher mean sleep duration (8.3 h) in their data. In our study sample, the mean sleep duration was 7.47 h, and it was similar among urban (7.48 h) and rural (7.51 h) residents. To examine this association more closely and to assess the impact of a healthy amount of sleep, we also conducted a sensitivity analysis for those with a healthy amount of sleep between 7 and 9 h. The associations observed for sleep remained statistically significant only among rural residents. Overall, these results highlight the importance of future studies addressing adequate sleep duration as a possible way to reduce depressive symptoms.

A clinically meaningful minimum reduction in BDI-II score has been suggested to be around 20%, depending on the severity of the symptoms [57, 58]. In our study, the decrease in the BDI-II score was generally around 5–10% for 30–60 min time reallocations, reaching − 13% at best when reallocating 60 min from LPA to sleep among rural residents. The percentages are, however, quite comparable to the ones reported by similar studies [12, 13] using questionnaire-based depressive symptoms as an outcome instead of clinical diagnosis. A previous meta-analysis suggested that the impact of PA on depression might be less pronounced in non-clinical populations compared to clinical populations [59].

Strengths of this study include the use of accelerometer-measured PA and ST, 24-h movement behaviour approach and a large, representative sample from a population-based cohort. The urban–rural classification of the residential environment was based on exact home coordinates. However, several limitations should be acknowledged. First, one of the main limitations of our study is the observational and cross-sectional design, which limits the inference of causality of associations. Due to the design, there is a possibility for reverse causation, and participants with depressive symptoms may be less active because of their symptoms. This means that instead of physical activity reducing depressive symptoms, it could be that depressive symptoms lead to reduced physical activity. Future studies with longitudinal design are needed to establish the causality of these associations, providing a more robust understanding of the underlying mechanisms. Second, reliance on self-reported sleep duration may introduce measurement error and bias, which may particularly affect the results for sleep duration and ST. Future studies using objective measures of sleep are warranted. Third, measuring PA using accelerometers also has some potential for error. Accelerometers do not capture extra load or the intensity of activity precisely if the body part where the sensor is attached stays still. There could be some misclassification, particularly between sitting and PA, or sleeping may be classified as ST (for example, in the case of napping during the day). Accelerometer data was collected more often during summer among rural residents, which might affect our results even after including the season of the data collection as a covariate. Fourth, although we adjusted the models for the strenuousness of work, we could not differentiate between leisure-time and work-related PA, or other domains of PA, which may have different associations with depressive symptoms. Fifth, as our study sample was homogenous in age and ethnicity, the generalisability of our findings may be limited to populations with similar demographic characteristics and environmental contexts. Depressive symptoms vary across different countries [60]; hence, some of the results may also be influenced by culture. In addition, our urban/rural classification is detailed in the context of Finland, which may differ from other countries as urban/rural definitions vary [61], thus making the comparison of studies difficult.

## Conclusions

Associations between 24-h movement behaviours and depressive symptoms differed according to urban/rural residency. For rural residents, higher time spent in MVPA or sleep was beneficial, whereas for urban residents higher time spent in ST in relation to other behaviours was unbeneficial. Residential environment, PA behaviour and sufficient amount of sleep should be considered in the prevention and treatment of depressive symptoms. The cross-sectional design of the study limits conclusions about causality of associations, and further research exploring the mechanisms underlying these associations in diverse populations and longitudinal study settings is warranted.

## Supplementary Information

Additional file 1: Table S1. Compositional means (percentages and minutes of a 24-h day) for MVPA, LPA, ST and sleep by urban/rural residency. Table S2. Compositional multiple linear regression estimates for BDI-II score by urban/rural residency, including participants with 7–9 h of sleep per day. Figure S1. The results of tests for U-shaped relationship between sleep duration and depressive symptoms (BDI-II score). A U-shaped relationship is present if the two lines have an opposite slope sign (b coefficient), and are individually significant (*p*<0.10). The vertical dashed line indicates the algorithmically selected breakpoint.

## Data Availability

NFBC data are available from the University of Oulu, Infrastructure for Population Studies. Permission to use the data can be applied for research purposes via an electronic material request portal. In the use of data, we follow the EU general data protection regulation (679/2016) and the Finnish Data Protection Act. The use of personal data is based on cohort participants’ written informed consent in their latest follow-up study, which may cause limitations to its use. Please contact the NFBC project center (NFBCprojectcenter(at)oulu.fi) and visit the cohort website (www.oulu.fi/nfbc) for more information.

## Abbreviations

BDI-II: Beck Depression Inventory-II
CI: Confidence interval
LPA: Light-intensity physical activity
MVPA: Moderate-to-vigorous-intensity physical activity
ST: Sedentary time
PA: Physical activity
SD: Standard deviation
NFBC1966: Northern Finland Birth Cohort
MAD: Mean amplitude deviation

## References

[1] World Health Organization. Depression and other common mental disorders: global health estimates. https://iris.who.int/bitstream/handle/10665/254610/WHO-MSD-MER-2017.2-eng.pdf?sequence=1. Accessed 11.3.2025.

[2] Marquez PV, Saxena S. Making mental health a global priority. Cerebrum Dana Forum Brain Sci. 2016;2016:cer-10 United States. PMC5198754 28058091

[3] Santomauro DF, Mantilla Herrera AM, Shadid J, Zheng P, Ashbaugh C, Pigott DM, et al. Global prevalence and burden of depressive and anxiety disorders in 204 countries and territories in 2020 due to the COVID-19 pandemic. Lancet. 2021;398(10312):1700–12. 10.1016/s0140-6736(21)02143-7. 34634250 10.1016/S0140-6736(21)02143-7 PMC8500697

[4] Mammen G, Faulkner G. Physical activity and the prevention of depression: a systematic review of prospective studies. Am J Prev Med. 2013;45(5):649–57. 10.1016/j.amepre.2013.08.001. 24139780 10.1016/j.amepre.2013.08.001

[5] Noetel M, Sanders T, Gallardo-Gómez D, Taylor P, Cruz B del P, Hoek D van den, et al. Effect of exercise for depression: systematic review and network meta-analysis of randomised controlled trials. BMJ. 2024;384:e075847. 10.1136/bmj-2023-075847. 10.1136/bmj-2023-075847 PMC10870815 38355154

[6] Singh B, Olds T, Curtis R, Dumuid D, Virgara R, Watson A, et al. Effectiveness of physical activity interventions for improving depression, anxiety and distress: an overview of systematic reviews. Br J Sports Med. 2023;57(18):1203–9. 10.1136/bjsports-2022-106195. 36796860 10.1136/bjsports-2022-106195 PMC10579187

[7] Bellón JÁ, Conejo-Cerón S, Sánchez-Calderón A, Rodríguez-Martín B, Bellón D, Rodríguez-Sánchez E, et al. Effectiveness of exercise-based interventions in reducing depressive symptoms in people without clinical depression: systematic review and meta-analysis of randomised controlled trials. Br J Psychiatry. 2021;219(5):578–87. 10.1192/bjp.2021.5. 33533706 10.1192/bjp.2021.5

[8] Zhai L, Zhang H, Zhang D. Sleep duration and depression among adults: a meta-analysis of prospective studies. Depress Anxiety. 2015;32(9):664–70. 10.1002/da.22386. 26047492 10.1002/da.22386

[9] Huang Y, Li L, Gan Y, Wang C, Jiang H, Cao S, et al. Sedentary behaviors and risk of depression: a meta-analysis of prospective studies. Transl Psychiatry. 2020;10(1):26. 10.1038/s41398-020-0715-z. 32066686 10.1038/s41398-020-0715-z PMC7026102

[10] Chastin SFM, Palarea-Albaladejo J, Dontje ML, Skelton DA. Combined effects of time spent in physical activity, sedentary behaviors and sleep on obesity and cardio-metabolic health markers: a novel compositional data analysis approach. PLoS ONE. 2015;10(10): e0139984. 10.1371/journal.pone.0139984. 26461112 10.1371/journal.pone.0139984 PMC4604082

[11] Blodgett JM, Mitchell JJ, Stamatakis E, Chastin S, Hamer M. Associations between the composition of daily time spent in physical activity, sedentary behaviour and sleep and risk of depression: compositional data analyses of the 1970 British cohort Study. J Affect Disord. 2023;320:616–20. 10.1016/j.jad.2022.09.110. 36183826 10.1016/j.jad.2022.09.110

[12] del Pozo CB, Alfonso-Rosa RM, McGregor D, Chastin SF, Palarea-Albaladejo J, del Pozo CJ. Sedentary behaviour is associated with depression symptoms: compositional data analysis from a representative sample of 3233 US adults and older adults assessed with accelerometers. J Affect Disord. 2020;265:59–62. 10.1016/j.jad.2020.01.023. 31959584 10.1016/j.jad.2020.01.023

[13] Kandola AA, del Pozo CB, Osborn DPJ, Stubbs B, Choi KW, Hayes JF. Impact of replacing sedentary behaviour with other movement behaviours on depression and anxiety symptoms: a prospective cohort study in the UK Biobank. BMC Med. 2021;19(1):133. 10.1186/s12916-021-02007-3. 34134689 10.1186/s12916-021-02007-3 PMC8210357

[14] United Nations. 68% of the world population projected to live in urban areas by 2050, says UN. 2018. https://www.un.org/development/desa/en/news/population/2018-revision-of-world-urbanization-prospects.html. Accessed 11.3.2025.

[15] Vlahov D, Galea S. Urbanization, urbanicity, and health. J Urban Heal. 2002;79(1):S1-12. 10.1093/jurban/79.suppl_1.S1. 10.1093/jurban/79.suppl_1.S1 PMC3456615 12473694

[16] Rautio N, Seppänen M, Timonen M, Puhakka S, Kärmeniemi M, Miettunen J, et al. Associations between neighbourhood characteristics, physical activity and depressive symptoms: the Northern Finland Birth Cohort 1966 Study. Eur J Public Health. 2024;34(1):114–20. 10.1093/eurpub/ckad215. 38081169 10.1093/eurpub/ckad215 PMC10843961

[17] Sampson L, Ettman CK, Galea S. Urbanization, urbanicity, and depression: a review of the recent global literature. Curr Opin Psychiatry. 2020;33(3):233–44. 10.1097/yco.0000000000000588. 32040041 10.1097/YCO.0000000000000588

[18] Rautio N, Filatova S, Lehtiniemi H, Miettunen J. Living environment and its relationship to depressive mood: a systematic review. Int J Soc Psychiatry. 2018;64(1):92–103. 10.1177/0020764017744582. 29212385 10.1177/0020764017744582

[19] Fan JX, Wen M, Kowaleski-Jones L. Rural–urban differences in objective and subjective measures of physical activity: findings from the National Health and Nutrition Examination Survey (NHANES) 2003–2006. Prev Chronic Dis. 2014;11: e141. 10.5888/pcd11.140189. 25144676 10.5888/pcd11.140189 PMC4149321

[20] Boakye K, Bovbjerg M, Schuna J, Branscum A, Varma RP, Ismail R, et al. Urbanization and physical activity in the global Prospective Urban and Rural Epidemiology study. Sci Rep. 2023;13(1):290. 10.1038/s41598-022-26406-5. 36609613 10.1038/s41598-022-26406-5 PMC9822998

[21] Kärmeniemi M, Lankila T, Ikäheimo T, Koivumaa-Honkanen H, Korpelainen R. The built environment as a determinant of physical activity: a systematic review of longitudinal studies and natural experiments. Ann Behav Med. 2018;52(3):239–51. 10.1093/abm/kax043. 29538664 10.1093/abm/kax043

[22] University of Oulu. Northern Finland birth cohort 1966. University of Oulu. 2024. http://urn.fi/urn:nbn:fi:att:bc1e5408-980e-4a62-b899-43bec3755243. Accessed 11.3.2025.

[23] Rantakallio P. The longitudinal study of the Northern Finland birth cohort of 1966. Paediatr Perinat Epidemiol. 1988;2(1):59–88. 10.1111/j.1365-3016.1988.tb00180.x. 2976931 10.1111/j.1365-3016.1988.tb00180.x

[24] Nordström T, Miettunen J, Auvinen J, Ala-Mursula L, Keinänen-Kiukaanniemi S, Veijola J, et al. Cohort profile: 46 years of follow-up of the Northern Finland Birth Cohort 1966 (NFBC1966). Int J Epidemiol. 2022;50(6):1786–7. 10.1093/ije/dyab109. 34999878 10.1093/ije/dyab109 PMC8743124

[25] Farrahi V, Kangas M, Walmsley R, Niemelä M, Kiviniemi A, Puukka K, et al. Compositional associations of sleep and activities within the 24-h cycle with cardiometabolic health markers in adults. Med Sci Sports Exerc. 2021;53(2):324–32. 10.1249/mss.0000000000002481. 32776775 10.1249/MSS.0000000000002481 PMC7879600

[26] Aittasalo M, Vähä-Ypyä H, Vasankari T, Husu P, Jussila A-M, Sievänen H. Mean amplitude deviation calculated from raw acceleration data: a novel method for classifying the intensity of adolescents’ physical activity irrespective of accelerometer brand. BMC Sport Sci Med Rehabil. 2015;7:18. 10.1186/s13102-015-0010-0 PMC4527117 26251724

[27] Vähä-Ypyä H, Husu P, Suni J, Vasankari T, Sievänen H. Reliable recognition of lying, sitting, and standing with a hip-worn accelerometer. Scand J Med Sci Sports. 2018;28(3):1092–102. 29144567 10.1111/sms.13017

[28] Choi L, Liu Z, Matthews CE, Buchowski MS. Validation of accelerometer wear and nonwear time classification algorithm. Med Sci Sports Exerc. 2011;43(2):357–64. 10.1249/mss.0b013e3181ed61a3. 20581716 10.1249/MSS.0b013e3181ed61a3 PMC3184184

[29] Vähä-Ypyä H, Vasankari T, Husu P, Mänttäri A, Vuorimaa T, Suni J, et al. Validation of cut-points for evaluating the intensity of physical activity with accelerometry-based mean amplitude deviation (MAD). PLoS ONE. 2015;10(8): e0134813. 10.1371/journal.pone.0134813. 26292225 10.1371/journal.pone.0134813 PMC4546343

[30] Finnish Environment Institute. YKR urban‐rural classification. 2010. https://ckan.ymparisto.fi/dataset/kaupunki-maaseutu-luokitus-ykr. Accessed 11.3.2025.

[31] Helminen V, Nurmio K, Rehunen A, Ristimäki M, Oinonen K, Tiitu M, et al. Kaupunki‐maaseutu‐alueluokitus. paikkatietoihin perustuvan alueluokituksen muodostamisperiaatteet. [urban‐rural‐classification. the basis for development of geographical information‐based area classification system]. 2014. http://hdl.handle.net/10138/135861. Accessed 11.3.2025.

[32] Beck AT, Steer RA, Brown GK. Manual for beck depression inventory-II. San Antonio, TX: Psychological Corporation; 1996.

[33] Punakallio A, Lusa S, Ala-Mursula L, Ek E, Nevanperä N, Remes J, et al. Personal meaning of work and perceived work ability among middle-aged workers with physically strenuous work: a Northern Finland Birth Cohort 1966 Study. Int Arch Occup Environ Health. 2019;92(3):371–81. 10.1007/s00420-019-01412-9. 30767053 10.1007/s00420-019-01412-9 PMC6420453

[34] Vladimirov D, Niemelä S, Keinänen-Kiukaanniemi S, Ala-Mursula L, Auvinen J, Timonen M, et al. Cloninger’s temperament dimensions and longitudinal alcohol use in early midlife: a Northern Finland Birth Cohort 1966 Study. Alcohol Clin Exp Res. 2018;42(10):1924–32. 10.1111/acer.13857. 30063251 10.1111/acer.13857

[35] Celikel FC, Kose S, Cumurcu BE, Erkorkmaz U, Sayar K, Borckardt JJ, et al. Cloninger’s temperament and character dimensions of personality in patients with major depressive disorder. Compr Psychiatry. 2009;50(6):556–61. 10.1016/j.comppsych.2008.11.012. 19840594 10.1016/j.comppsych.2008.11.012

[36] O’Connell SE, Griffiths PL, Clemes SA. Seasonal variation in physical activity, sedentary behaviour and sleep in a sample of UK adults. Ann Hum Biol. 2014;41(1):1–8. 10.3109/03014460.2013.827737. 23992280 10.3109/03014460.2013.827737

[37] Gupta N, Mathiassen SE, Mateu-Figueras G, Heiden M, Hallman DM, Jørgensen MB, et al. A comparison of standard and compositional data analysis in studies addressing group differences in sedentary behavior and physical activity. Int J Behav Nutr Phys Act. 2018;15(1):53. 10.1186/s12966-018-0685-1. 29903009 10.1186/s12966-018-0685-1 PMC6003121

[38] Chastin SFM, Palarea-Albaladejo J, Dontje ML, Skelton DA. Combined effects of time spent in physical activity, sedentary behaviors and sleep on obesity and cardio-metabolic health markers: a novel compositional data analysis approach. PLoS One. 2015;10(10): e0139984. 10.1371/journal.pone.0139984. 26461112 10.1371/journal.pone.0139984 PMC4604082

[39] Dumuid D, Stanford TE, Martin-Fernández JA, Pedišić Ž, Maher CA, Lewis LK, et al. Compositional data analysis for physical activity, sedentary time and sleep research. Stat Methods Med Res. 2018;27(12):3726–38. 10.1177/0962280217710835. 28555522 10.1177/0962280217710835

[40] Wang L, He S, Yan N, Pan R, Niu Y, Li J. Mediating role of depressive symptoms on the relationship between sleep duration and cognitive function. Sci Rep. 2023;13(1):4067. 10.1038/s41598-023-31357-6. 36906644 10.1038/s41598-023-31357-6 PMC10008529

[41] Simonsohn U. Two Lines: A Valid Alternative to the Invalid Testing of U-Shaped Relationships With Quadratic Regressions. Adv Methods Pract Psychol Sci. 2018;1(4):538–55. 10.1177/2515245918805755.

[42] Hirshkowitz M, Whiton K, Albert SM, Alessi C, Bruni O, DonCarlos L, et al. National Sleep Foundation’s updated sleep duration recommendations: final report. Sleep Heal. 2015;1(4):233–43. 10.1016/j.sleh.2015.10.004. 10.1016/j.sleh.2015.10.004 29073398

[43] R Core Team. R: A language and environment for statistical computing. Vienna, Austria: R Foundation for Statistical Computing; 2022. https://www.r-project.org/. Accessed 11.3.2025.

[44] Posit Team. RStudio: integrated development environment for R. Boston, MA: Posit Software, PBC; 2023. http://www.posit.co/. Accessed 11.3.2025.

[45] Templ M, Hron K, Filzmoser P. robCompositions: an R-package for robust statistical analysis of compositional data. In: Compositional data analysis: theory and applications. John Wiley and Sons; 2011. p. 341–55.

[46] van den Boogaart KG, Tolosana-Delgado R, Bren M. compositions: compositional data analysis. 2023. https://cran.r-project.org/package=compositions. Accessed 11.3.2025.

[47] Wickham H, Averick M, Bryan J, Chang W, McGowan LD, François R, et al. Welcome to the tidyverse. J Open Source Softw. 2019;4(43):1686. 10.21105/joss.01686.

[48] Baptiste A. gridExtra: miscellaneous functions for “grid” graphics. 2017. https://cran.r-project.org/package=gridExtra. Accessed 11.3.2025.

[49] Rich B. table1: tables of descriptive statistics in html. 2023. https://cran.r-project.org/package=table1. Accessed 11.3.2025.

[50] Kandola A, Ashdown-Franks G, Hendrikse J, Sabiston CM, Stubbs B. Physical activity and depression: Towards understanding the antidepressant mechanisms of physical activity. Neurosci Biobehav Rev. 2019;107:525–39 https://www.sciencedirect.com/science/article/pii/S0149763419305640. 31586447 10.1016/j.neubiorev.2019.09.040

[51] Arent SM, Walker AJ, Arent MA. The effects of exercise on anxiety and depression. In: Handbook of sport psychology. 2020. p. 872–90. 10.1002/9781119568124.ch42.

[52] Füzéki E, Engeroff T, Banzer W. Health benefits of light-intensity physical activity: a systematic review of accelerometer data of the National Health and Nutrition Examination Survey (NHANES). Sport Med. 2017;47(9):1769–93. 10.1007/s40279-017-0724-0. 10.1007/s40279-017-0724-0 28393328

[53] Farrahi V, Kangas M, Kiviniemi A, Puukka K, Korpelainen R, Jämsä T. Accumulation patterns of sedentary time and breaks and their association with cardiometabolic health markers in adults. Scand J Med Sci Sports. 2021;31(7):1489–507. 10.1111/sms.13958. 33811393 10.1111/sms.13958

[54] Farrahi V, Rostami M, Dumuid D, Chastin SFM, Niemelä M, Korpelainen R, et al. Joint profiles of sedentary time and physical activity in adults and their associations with cardiometabolic health. Med Sci Sports Exerc. 2022;54(12):2118–28. 10.1249/mss.0000000000003008. 35881930 10.1249/MSS.0000000000003008 PMC9671590

[55] White RL, Babic MJ, Parker PD, Lubans DR, Astell-Burt T, Lonsdale C. Domain-specific physical activity and mental health: a meta-analysis. Am J Prev Med. 2017;52(5):653–66. 28153647 10.1016/j.amepre.2016.12.008

[56] Koohsari MJ, Sugiyama T, Sahlqvist S, Mavoa S, Hadgraft N, Owen N. Neighborhood environmental attributes and adults’ sedentary behaviors: review and research agenda. Prev Med. 2015;77:141–9. 10.1016/j.ypmed.2015.05.027. 26051198 10.1016/j.ypmed.2015.05.027

[57] Kounali D, Button KS, Lewis G, Gilbody S, Kessler D, Araya R, et al. How much change is enough? Evidence from a longitudinal study on depression in UK primary care. Psychol Med. 2022;52(10):1875–82. 10.1017/s0033291720003700. 33138872 10.1017/S0033291720003700 PMC9340848

[58] Button KS, Kounali D, Thomas L, Wiles NJ, Peters TJ, Welton NJ, et al. Minimal clinically important difference on the Beck Depression Inventory -II according to the patient’s perspective. Psychol Med. 2015;45(15):3269–79. 10.1017/s0033291715001270. 26165748 10.1017/S0033291715001270 PMC4611356

[59] Rebar AL, Stanton R, Geard D, Short C, Duncan MJ, Vandelanotte C. A meta-meta-analysis of the effect of physical activity on depression and anxiety in non-clinical adult populations. Health Psychol Rev. 2015;9(3):366–78. 10.1080/17437199.2015.1022901. 25739893 10.1080/17437199.2015.1022901

[60] Seppänen M, Lankila T, Auvinen J, Miettunen J, Korpelainen R, Timonen M. Cross-cultural comparison of depressive symptoms on the Beck Depression Inventory-II, across six population samples. BJPsych Open. 2022;8(2): e46. 10.1192/bjo.2022.13. 35144711 10.1192/bjo.2022.13 PMC8867877

[61] Krabbendam L, van Vugt M, Conus P, Söderström O, Abrahamyan Empson L, van Os J, et al. Understanding urbanicity: how interdisciplinary methods help to unravel the effects of the city on mental health. Psychol Med. 2021;51(7):1099–110. 10.1017/s0033291720000355. 32156322 10.1017/S0033291720000355

