# Supplementary material for: Compositional associations of 24-h physical activities, sedentary time and sleep with depressive symptoms in urban and rural residents: a cross-sectional study
Source: BMC Med. 2025 Apr 14;23:219. doi: 10.1186/s12916-025-04051-9 (PMC11995539; doi:10.1186/s12916-025-04051-9)
Supplement: Supplementary file 1 — Additional file 1: Table S1. Compositional means (percentages and minutes of a 24-h day) for MVPA, LPA, ST and sleep by urban/rural residency. Table S2. Compositional multiple linear regression estimates for BDI-II score by urban/rural residency, including participants with 7–9 h of sleep per day. Figure S1. The results of tests for U-shaped relationship between sleep duration and depressive symptoms (BDI-II score). A U-shaped relationship is present if the two lines have an opposite slope sign (b coefficient), and are individually significant (p<0.10). The vertical dashed line indicates the algorithmically selected breakpoint. [file 12916_2025_4051_MOESM1_ESM.docx]

**Table S1**. Compositional means (percentages and minutes of a 24-h day) for MVPA, LPA, ST and sleep by urban/rural residency.

|  | **MVPA** | **LPA** | **ST** | **Sleep** |
| --- | --- | --- | --- | --- |
| All (n=4,295) | 3.1% (45) | 26.6% (383) | 34.3% (494) | 35.9% (517) |
| Urban residents (n=2,868) | 3.2% (46) | 25.7% (370) | 35.2% (507) | 35.9% (517) |
| Rural residents (n=1,427) | 3.0% (43) | 28.5% (410) | 32.5% (468) | 36.0% (518) |

*MVPA, moderate-to-vigorous-intensity physical activity; LPA, light-intensity physical activity; ST, sedentary time.*

**Table S2**. Compositional multiple linear regression estimates for BDI-II score by urban/rural residency, including participants with 7–9 h of sleep per day.

|  | **Model R2** | **Model p** | **MVPA β (95% CI)** | **p** | **LPA β (95% CI)** | **p** | **ST β (95% CI)** | **p** | **Sleep β (95% CI)** | **p** |
| --- | --- | --- | --- | --- | --- | --- | --- | --- | --- | --- |
| All (n=2,874) | 0.21 | **<0.001** | -0.18 (-0.59–0.22) | 0.374 | 0.18 (-0.83–1.19) | 0.731 | 0.21 (-0.92–1.34) | 0.715 | -0.20 (-2.03–1.62) | 0.827 |
| Urban residents (n=1,953) | 0.22 | **<0.001** | -0.11 (-0.62–0.40) | 0.679 | -0.93 (-2.20–0.33) | 0.149 | -0.54 (-1.97–0.90) | 0.455 | 1.58 (-0.69–3.86) | 0.172 |
| Rural residents (n=921) | 0.23 | **<0.001** | -0.39 (-1.07–0.28) | 0.251 | **2.76 (1.09–4.44)** | **0.001** | **1.93 (0.08–3.79)** | **0.041** | **-4.30 (-7.35– -1.25)** | **0.006** |

Significant associations at level p<0.05 are shown in bold and associations at level p<0.10 in italic. All of the models have been adjusted with sex, education, employment status, strenuousness of work, marital status, harm avoidance personality trait score, alcohol consumption, smoking status, waist circumference and the season of the PA data collection.

*MVPA, moderate-to-vigorous-intensity physical activity; LPA, light-intensity physical activity; ST, sedentary time; CI, confidence interval.*

**
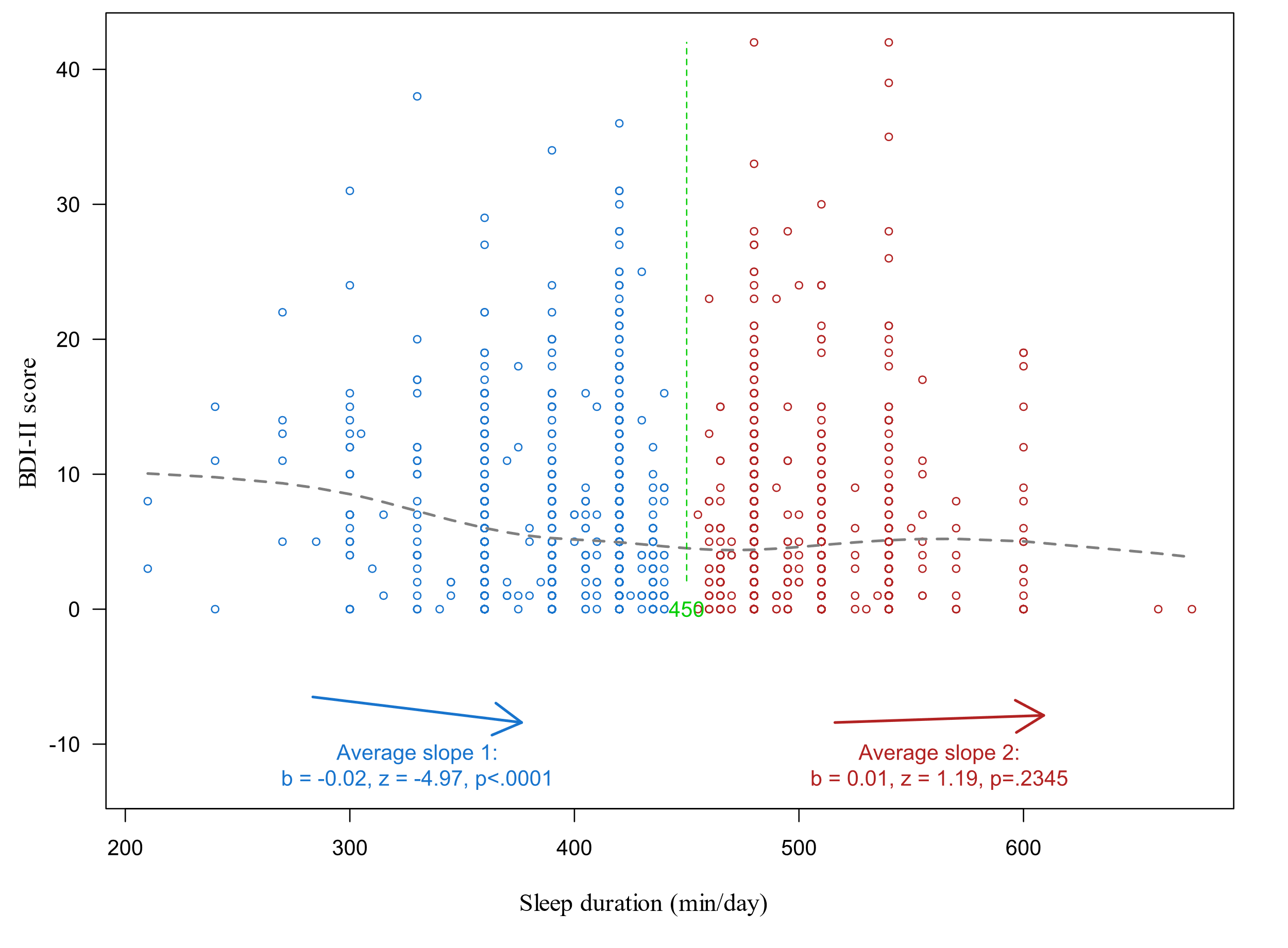
**

**Figure S1.** The results of tests for U-shaped relationship between sleep duration and depressive symptoms (BDI-II score). A U-shape relationship is present if the two lines have an opposite slope sign (b coefficient), and are individually significant (p<0.10). The vertical dashed line indicates the algorithmically selected breakpoint.

*BDI-II, Beck Depression Inventory II*
